# Potential of Gene and Cell Therapy for Inner Ear Hair Cells

**DOI:** 10.1155/2018/8137614

**Published:** 2018-06-13

**Authors:** Min Yong Lee, Yong-Ho Park

**Affiliations:** ^1^Department of Otorhinolaryngology and Head & Neck Surgery, Dankook University Hospital, Cheonan, Chungnam, Republic of Korea; ^2^Department of Otolaryngology-Head and Neck Surgery, College of Medicine, Chungnam National University, Daejeon, Republic of Korea; ^3^Brain Research Institute, College of Medicine, Chungnam National University, Daejeon, Republic of Korea

## Abstract

Sensorineural hearing loss is caused by the loss of sensory hair cells (HCs) or a damaged afferent nerve pathway to the auditory cortex. The most common option for the treatment of sensorineural hearing loss is hearing rehabilitation using hearing devices. Various kinds of hearing devices are available but, despite recent advancements, their perceived sound quality does not mimic that of the “naïve” cochlea. Damage to crucial cochlear structures is mostly irreversible and results in permanent hearing loss. Cochlear HC regeneration has long been an important goal in the field of hearing research. However, it remains challenging because, thus far, no medical treatment has successfully regenerated cochlear HCs. Recent advances in genetic modulation and developmental techniques have led to novel approaches to generating HCs or protecting against HC loss, to preserve hearing. In this review, we present and review the current status of two different approaches to restoring or protecting hearing, gene therapy, including the newly introduced CRISPR/Cas9 genome editing, and stem cell therapy, and suggest the future direction.

## 1. Background

Hearing loss can be divided into sensorineural and conductive hearing loss. Conductive hearing loss is a biophysical problem, resulting from the fixation or disruption of the ossicular chain, middle ear effusion, and third window of the cochlea. In most patients these problems can be surgically managed. By contrast, sensorineural hearing loss is caused by the loss of sensory hair cells (HCs) or damage involving the afferent nerve pathway to the auditory cortex. These types of damage are caused by a variety of ototoxic agents, such as aminoglycoside and cisplatin, acoustic overexposure, and mutations in the genes responsible for hearing and aging. They are mostly irreversible and result in permanent hearing loss.

The current clinical option for sensorineural hearing loss is hearing rehabilitation with hearing devices, which range from externally worn to implantable devices. Yet, despite recent advances in hearing aid and cochlear implant technologies, the perceived sound quality does not mimic that of the “naïve” cochlea. Impaired speech perception in noisy environments and musical sound perception are well-known drawbacks of cochlear implantation [1, 2] and representative of the inability of current technologies to completely reproduce the unique and complex functions of HCs that allow sound perception.

HC regeneration is one of the most important goals in the field of hearing research. In the past two decades, differences in HC characteristics among species and between sensory organs have been explored. Unlike mammalian HCs, the HCs of avian species [3] regenerate if lost. In addition, the regenerative potential of fatally damaged vestibular HCs has been demonstrated [4]. Recognition of the key features of avian and vestibular HCs may provide insights into new forms of hearing loss therapy. For example, technical advances in genetic modulation and development could be used to determine the factors needed for HC regeneration, the expression of which could then be genetically modified to regenerate HCs or their precursor supporting cells (SCs). An alternative approach would be to use newly identified factors to generate HCs from implanted stem cells.

Because exposure to ototoxic and acoustic insults is sometimes unavoidable, protecting HCs from possible ototoxic insult has also been considered, and drugs able to prevent hearing loss related to various ototoxic insults have been studied but, thus far, without clinical success [5–10], one difficulty is drug delivery to the cochlear HCs and the achievement of high drug concentrations at the time of ototoxic exposure. Thus, a better strategy may be to reprogram the cells so that they have the potential to protect themselves.

**Figure 1:**
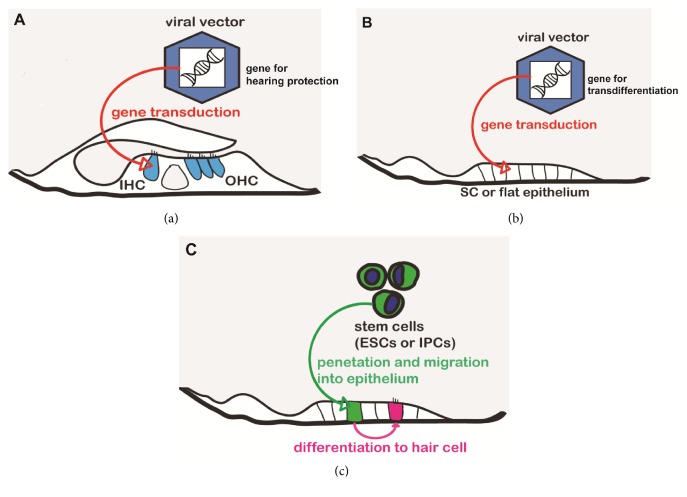
**Gene and stem cell therapies for hearing loss**. Viral vectors carrying a protective gene are delivered into the fluid cavity of the cochlea, where they transfect hair cells and ultimately protect hearing (a). The regeneration of hair cells is achieved by the transduction of supporting cells of the flat epithelium using a viral vector carrying a regenerative gene (b). In stem cell therapy, pluripotent stem cells are delivered into the fluid-filled cochlear cavity and then migrate and penetrate into the flat epithelium. With the help of growth factors, these cells will differentiate into the hair-cell-like cells (c) (OHC: outer hair cell, IHC: inner hair cell, SC: supporting cell, ESC: embryonic stem cell, and IPC: induced pluripotent cell).

In this review, we introduce two different approaches to restoring or protecting hearing. The first is gene therapy (Figures 1(a) and 1(b)), in which viral vectors, siRNA, or similar agents are used to specifically modulate the expression of genes necessary for HC regeneration or protection. The second is stem-cell therapy (Figure 1(c)), in which cells capable of differentiating into HCs, such as induced pluripotent cells (IPCs) or embryonic stem cells (ESCs), are forced to differentiate into HCs by exposure to the responsible factors.

## 2. Gene Therapy for Hearing Loss

### 2.1. Introduction

The history of gene therapy began in the 1960s and early 1970s, when genetically marked cells were developed and used to understand the mechanism of cellular transformation by several viral vectors. With the introduction of, and advances in, recombinant DNA techniques and gene cloning, cell-based trials demonstrated the possibilities of repairing defective genes in vitro. The development of retroviral vectors and other gene transduction methods allowed for more efficient phenotype corrections in animal models [12]. Following these successful studies, gene therapy has been applied in numerous fields in medicine, from neural cell regeneration to anticancer therapy. Clinical trials using gene therapy have been conducted for the treatment of Alzheimer's disease [13], pancreatic cancer [14], muscular disease, [15] and eye diseases [16–18]. More recently, for the first time in the USA, the FDA approved the use of gene therapy for acute lymphoblastic leukemia (https://www.fda.gov/NewsEvents/Newsroom/PressAnnouncements/ucm574058.htm).

Nevertheless, in the treatment of hearing loss, gene therapy must overcome several obstacles related to the characteristics and anatomy of the structures of the inner ear. The cochlea is a closed, fluid-filled cavity covered by bone, and it is very vulnerable to changes in the amount and composition of inner ear fluid. Consequently, the delivery of therapeutic materials into the cochlea without damaging homeostasis is very challenging. A possible route is the round window, which is the only membranous structure covering the cochlea and the exit site of the wave traveling from the oval window (point of connection to the ossicles). Another option is to insert material inside the cochlear cavity to create an opening, in a procedure called a cochleostomy. This was the approach used by our group to inject material into the three cochlear cavities (scala vestibule, scala media, and scala tympani), because via the round window only the scala tympani is accessible.

Viral vectors can be used to deliver a gene or small molecule able to modulate gene expression into target cells in the cochlea. The viral vectors most commonly used for this purpose are adenovirus (Adv) and adenoassociated virus (AAV). Other candidates such as retrovirus and lentivirus were also used but were relatively less safe and effective [19]. For use in the ear, AAV vectors are often preferred because of their safety and selective activity for several types of specialized cells in the inner ear [20, 21]. There are 12 AAV vectors with various serotypes (AAV1, AAV2, AAV5, AAV6, AAV6.2, AAV7, AAV8, AAV9, rh.8, rh.10, rh.39, and rh.43) and it was reported that there is different target cell (among cochlea) specificity among serotypes [20]. AAV1, AAV8, and AAV9 have shown their specific preference to inner hair cell of adult mice and AAV2, AAV5, and rh.10 have shown their specific preference to inner hair cell of neonatal mice. Furthermore AAV vector technologies have remarkably advanced during the past few years, mutating the AAV virus to enhance their effectivity and safety for each specific use. One good example for this is Anc80L65 which is a tailored and revolutionized AAV vector. Using this vector for gene delivery to the organ of Corti resulted in an extremely efficient outcome showing viral transduction from inner hair cells to very last row of outer hair cells and extending to the vestibular organs as well as to the cochlea (apex to base) with minimal damage of resident cells [21, 22].

The discovery of RNA interference (RNAi) which is a natural process of knocking down the targeted gene presents an alternative method to modulate gene expression [23]. There are two types of RNAi application molecule, small interfering RNA (siRNA) which is chemically synthesized double-stranded RNA [24] and short hairpin RNA (shRNA) which is vector based RNA [25]. Several studies have reported protection against ototoxic insults* in vitro* and* ex vivo* using RNAi [26–29]. Despite the successful delivery of siRNA to the cochlea [30, 31], not many studies were successful in translating the outcome to* in vivo* studies. The areas of research using viral vector gene therapy or RNAi can be divided into genetic hearing loss, hearing protection, and HC regeneration. Recently, more efficient targeted genome editing by clustered regularly interspaced short palindromic repeats (CRISPR)/CRISPR-associated nuclease 9 (Cas9) technology has been introduced and will be discussed in detail in the following sections.

### 2.2. Gene Therapy for Genetic Hearing Loss

The screening, diagnosis, and rehabilitation of genetic congenital hearing loss have become well established, to the benefit of patients and, in affected children, their parents. With the aid of a hearing aid and cochlear implant, patients with genetic congenital hearing loss can receive proper education and care. However, as noted above, currently available devices do not completely replicate the quality of sound received by the naïve ear, and the use of the electronic device is cumbersome. Genetic mutations resulting in hearing loss can already be screened for in utero. Ideally, a genetic mutation involving hearing loss that is found before birth would be treated by replacing the defective gene by gene therapy. In utero gene delivery has been achieved in animals and some studies have shown that cure was achieved. Methionine sulfoxide reductase B3 (MsrB3) is an important protein for auditory function in mammals. Its depletion leads to the degeneration of stereociliary bundles and the death of HC, resulting in severe congenital hearing loss. Delivery of the MsrB3 gene using AAV virus directly to the otocyst at E12/5 rescued hearing function after birth [32].

One of the common gene mutations related to human genetic hearing loss involves GJB2, which encodes connexin 26, a gap junction protein that enables the recycling of toxic material such as potassium. The lack of connexin results in the accumulation of potassium in the extracellular fluid and thus a decrease in endocochlear potential, eventually causing hearing damage. Using an animal model of the GJB2 mutation, Yu et al. were able to restore the depleted connexin by gene therapy [33]; however, hearing function remained poor, which demonstrated the necessity for early intervention. Indeed, early intervention using an AAV vector carrying the Gjb2 gene in a genetically mutated mouse, before the onset of damage, allowed the preservation of hearing [34]. The results of that study indicated that this mutation causes hearing loss via different pathophysiologic mechanisms. Studies of other parts of the inner ear, specifically, in the vestibular organ, showed that a loss of connexin 26 does not affect the balance function in mice with a GJB2 mutation [35]. Thus, there may be a compensatory mechanism for the vestibular organ, the elucidation of which might contribute to curing this most common type of genetic hearing loss.

Mice with VGLUT3 mutations lack vesicular glutamate transporters and exhibit hearing loss by disrupted synaptic transmission. Studies in humans suggested that missense mutations in the human gene SLC17A8 (encoding VGLUT3) are related to the progressive loss of hearing at high frequencies, as observed in DFNA25 mutations. In an animal model of hearing loss related to a VGLUT3 mutation, histologic and functional recovery of hearing was achieved when gene therapy using AAV was administered before the maturation of hearing function [36]. Similar outcome of gene therapy showing recovery of phenotype by delivery of gene using AAV vector was observed in animal model for Usher syndrome [37] and genetic hearing loss related to human DFNB7/11 and DFNA36 [38]. Recently using the advanced synthetic AAV (Anc80L65, described above) has also resulted in rescue of the phenotype from Ush1c mutation [39].

These very promising outcomes (Table 1) suggest the potential of gene therapy in patients with genetic hearing loss, when the mutation and its imposed functional deficit are well characterized and evident. With increasing knowledge of the genetics of hearing loss, the clinical application of gene therapy may not be far away.

### 2.3. Gene Therapy for Hearing Preservation

In modern society, ototoxic exposure, for example, to aminoglycosides and cisplatin, as well as acoustic overexposure, is commonplace. Although many pharmacologic agents have been investigated, none were shown to be clinically useful in preventing ototoxicity. The advantage of gene therapy over pharmacological approaches is the prolonged expression of the gene and, therefore, of the therapeutic agent. A serious drawback is the inevitable damage of sensory cells during the surgical process. Recent studies using empty AAV vectors in cochlear HCs demonstrated the potential safety of the procedure but further studies with actual vectors carrying the required gene are still needed.

The molecules exhibiting protective effects against aminoglycosides when delivered by gene therapy are neurotrophins [40–42], blc2 [43], Hsp70 [44], and superoxide dismutase 1 [45], all of which increase the survival of HCs. In protecting against the ototoxicity of cisplatin, overexpression of the X-linked inhibitor of apoptosis, transient receptor potential vanilloid 1, and STAT via gene therapy was shown to be effective [46–50] (Table 2). By contrast, despite its higher incidence and the economic consequences, there has been little success in preventing hearing loss caused by acoustic overexposure. Neurotrophin 3 [51], with the potential to resolve the synaptopathy caused by overstimulation, may be a candidate, but noninvasive methods to deliver the Nt3 gene to the cochlea before acoustic ototoxicity should be investigated before extensive gene therapy studies are conducted. Recently, inhibition of AMP-activated protein kinase (AMPK) by siRNA has shown a protective effect against acoustic overexposure in mouse [52], suggesting the alternative target for a therapeutic approach.

### 2.4. Gene Therapy for Hair Cell Regeneration

In inner ear research, the ultimate goal is HC regeneration. Unlike other epithelial cells, which consistently turn over and are replaced once lost or damaged, the HCs of the mammalian cochlea do not regenerate. Theoretically, there are two ways to force HC regeneration: by inducing either proliferation of the auditory epithelium or the direct transdifferentiation of SCs cell to HCs. Using transgenic mice lacking P27 cells [53–55] or Rb1 [56], newly generated hair cells and the proliferation of SCs were observed but whether the cells were functional was unclear.

Transdifferentiation, in which a cell phenotype is converted from one to another, has been previously described in the basilar papilla of birds [57, 58], the vestibular sensory system [59], and Barrett's metaplasia [60]. Successful HC regeneration was accomplished by taking advantage of the cellular potential for transdifferentiation. For example, a viral vector was used to overexpress Atoh1, a gene that is fundamental in the developmental differentiation of HCs. Ectopic HCs possibly generated by the transdifferentiation from SCs [61] or by the inhibition of SC-encoding genes were obtained. The transdifferentiation of SC to HC was also induced using a *γ*-secretase inhibitor to block notch signaling [62]. A combined approach also resulted in HC regeneration [63]. Taken together, these findings (Table 3) showed that, by modulating specific genes, HC regeneration may be possible; however, this approach must first be optimized before it can be clinically applied in deaf patients.

### 2.5. CRISPR/Cas9 for Hearing Research

This approach is designed based on the protective mechanism of bacteria against viral infection and it is the most recent and advanced among the three programmable nucleases adapted for genome engineering [64]. With the benefits (simple design and fewer off-target effects) over previous gene regulating methods such as RNAi and other programmable nucleases, use of this technique is increasing rapidly in the field of hearing research. Recent applications are focused on generation of transgenic mouse to model the human hearing loss [65–70] using the technique which is less time-consuming and labor intensive compared to traditional methods. Trials to facilitate therapeutic gene delivery application are conducted in animal model. Efficient disruption of GFP (or any future target gene) expression was achieved using a modification of the current CRISPR/Cas9 system [71]. In addition, postponing the age related hearing loss was reported in conjunction with homology directed repair technique in mouse model by modulating the possible gene [39]. Therapeutic application for dominant syndromic disorder such as Usher syndrome would be a good candidate disease for the CRISPR/cas9 technology since it requires effective genome editing for the large size of gene mutation which gives less opportunity for AAV gene therapy. Recent publication pushed us a little bit closer to the ultimate goal, describing a successful* in vitro* mutation repair using homologous recombination [72]. However, there still remain some challenges for direct application of this technique for clinical use such as requirement of a protospacer-adjacent motif (PAM) since editing is only possible at the site in which Cas9 recognizes PAM [64]. However, rapid technological advancement during the past few years has shown great promise for development of new types of treatments for intractable diseases such as sensorineural hearing loss.

## 3. Stem Cell Therapy for Hearing Loss

### 3.1. Introduction

Stem cells are widely used in the field of regenerative medicine. These undifferentiated biological cells either differentiate into specialized cells or divide by mitosis to produce more stem cells. Both ESCs and induced pluripotent stem cells can be used in the regeneration of specific cells and tissue, as is required when damage to a tissue or other anatomic structure has occurred and self-regeneration is not possible.

The use of stem cells in hearing loss therapy has several prerequisites. First, since HCs are not easy to regenerate in the mature mammalian cochlea, whether they can be produced by stem cells is unclear. Second, due to the hostile environment of the cochlea itself, the high potassium level of the endolymph, and the presence of tight junctions that block insertion of the implanted cells into the organ of Corti [73], survival of the implanted stem cells or otic precursor cells is not guaranteed. There are two approaches for the delivery of exogenous cell: one is into the scala tympani through the round window or cochleostomy and inducing them to migrate into the organ of Corti [67]; the alternative is injecting directly into the scala media and optimizing the survival of the implanted cell which also involves adaptation of the transplantation surgery procedure.

In the following, we introduce the techniques shown to generate HCs from stem cells and discuss the conditioning methods that can increase the survival of transplanted stem cells in the scala media.

### 3.2. Differentiation of Stem Cells into Cochlear Hair Cell

There are several published protocols to generate the hair-cell-like cell from pluripotent stem cells [74–79]. In majority of these protocols, the successful differentiation rate was relatively low, only 1 or 2% of total cells were differentiated into the target cells [77]. Recent approaches that are adopting 3D culture technique successfully differentiated hair cells from embryonic stem cell, both rodent and human [76, 80–83]. In this section we are trying to explain the differentiation protocol by adopting well established 3D culture technique in prior publication [81].

**Figure 2:**
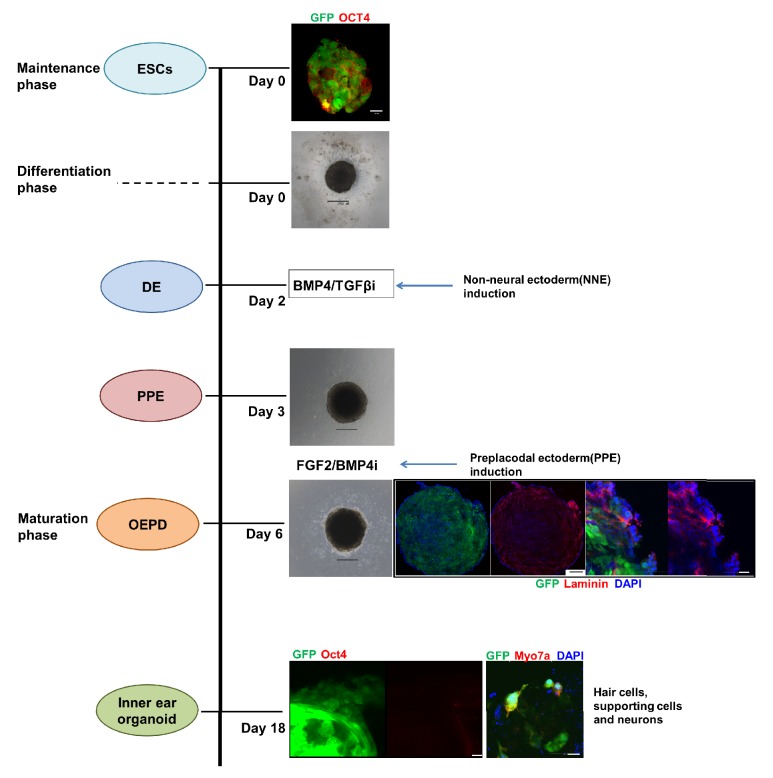
**Differentiation of the inner ear organoid over time**. The morphology and differentiation patterns of stem cells at different stages over time are shown. Differentiation is divided into three phases (maintenance, differentiation, and maturation) and can be followed by epifluorescence microscopy. The hanging drop technique was used for embryonic body (EB) formation. In the differentiation phase, BPM4 and TGF*β*i are administered on day 2 to induce nonneurodermal ectoderm as well as FGF2 and BPM4i on day 3 to induce preplacodal ectoderm. Day 6 marks the beginning of the maturation phase. The cells were fixed for microscopic analysis on day 18, at which time formation of the organoid structure was observed. These organoids contain several Myo7a-positive cells. ESCs, embryonic stem cells; EB embryonic body; DE, definitive ectoderm; OEPD, otic-epibranchial placode domain. White scale bars are 20 um. Black scale bars are 250 um.

The differentiation of HCs from embryonic stem cells takes place in three different phases (Figure 2). During the maintenance phase, the stem cells proliferate enough to form an embryonic body. The application of several growth factors and inhibitors to this embryonic body makes it start to differentiate, first into nonneurodermal ectoderm, then into preplacodal ectoderm, and eventually to otic placode. Among the key factors needed for this differentiation phase is fibroblast growth factor. The differentiated otic placode is then transferred to maturation medium, where it matures over a period of 10–15 days, marking the maturation phase. The resulting inner ear organoid was shown in previous reports to exhibit both functional activity and connecting nerve fibers [80–82].

Although the differentiation of stem cells to HCs seems to be well established, based on literature reports, a careful step by step reevaluation of the differentiation process is needed to allow its optimization and to minimize potential complications when the method is clinically applied. There are efforts to reduce the steps and to increase the efficiency for the differentiation process by gene modulation [76, 77]. Despite early stage of technology to generate the inner ear organoid, these approaches might shed light on design of a therapeutic modality for clinical stem cell application for hearing loss patients.

### 3.3. Enhancement of Stem Cell Survival after Intracochlear Transplantation

Given the above described information, very hostile environment of the endolymph (fluid cavity inside scala media), including the high potassium concentration and tight junction barrier[73], the survival of foreign cells is very limited. ESCs targeted for HC differentiation are histologically unstable and less robust than, for example, hilar cells. Therefore, the use of stem cells must be accompanied by methods that make the endolymphatic environment more hospitable. An alternative way is to deliver the stem cells to the scala tympani and guiding the cells to migrate into the target area [67]. But to do so with high efficiency and to minimize the undesirable effect in nontarget areas, strategies for efficient homing are required.

Here we would like to discuss more about the former option and introduce ways to design less hostile cochlea environment. There are several ways to reduce the high potassium content. One is simply flushing out the scala media fluid and replacing its content with one more hospitable to stem cells, such as stem cell media. Another is the administration of a systemic potassium-lowering drug, such as loop diuretics.

These two techniques were used in HeLa cells [84], which are more robust than stem cells and survived for at least 7 days following treatment. While the successful survival of human embryonic stem cells was also reported, it was not maintained for >1 day [85]. More favorable outcome was shown to be feasible when an additional conditioning procedure, the addition of sodium caprate, was included [86, 87]. Sodium caprate results in a temporary disruption of the tight junctions of the auditory epithelium. When used together with a potassium-lowering method, sodium caprate supported transplanted stem cell survival for at least seven days [85].

Although these results might be applicable for animals in acute model, there is a possibility that these processes might not be necessary in chronic condition which is more similar to the clinical hearing loss circumstances. It is not obvious whether high potassium concentration will remain after the loss of the crucial structures. Further clarification of ionic composition of scala media after the long duration deafness might minimize the necessity of conditioning procedure.

### 3.4. Future Directions

**Figure 3:**
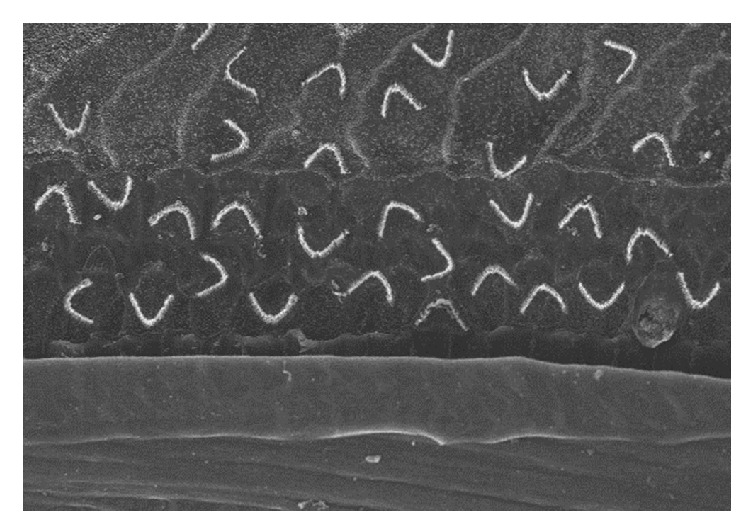
**Virtual features of disorganized hair cells positioning which possibly derived from transplanted stem cells or regeneration (adopted from Park et al.; see citation [11])**. Disorganized V-shaped structures of stereocilia bundles are observed at sites where supporting cells should reside. The image is based on the hypothesis that transplanted stem cells randomly placed around the organ of Corti will differentiate into outer HCs.

Many new scientific developments can be expected when the in vitro differentiation of stem cells into HCs is combined with in vivo alterations that lead to an environment hospitable for stem cells to survive long enough for their clinical use. Whether fully differentiated HCs can be obtained [11] in the deafened cochlea injected with stem cells from the outside (Figure 3) is unclear. If so, it will likely require support from other techniques, such as the damage-free injection of the required growth factors or inhibitors into the cochlea using, for example, a mini-osmotic pump. Innervating the cells with nerve fibers is also necessary for a positive functional outcome and might be feasible using neurotrophin gene therapy [51, 88–90], which has already been widely tested in nerve regeneration and growth.

Alternatively, we could expand our interest to the cochlear implantees with expected poor outcome due to the degeneration of nervous tissue [91]. With higher number of cell populations in Rosenthal's canal by transplantation of neural stem cells induced from embryonic cells or from differentiated cells [92–97], there might be a better performance of cochlear implant leading to increase of quality of sound perception. Still connection between the transplanted spiral ganglion cells and cochlear nucleus has to be confirmed prior to clinical applications.

## 4. Limitations and Conclusion

One of the limitations of using AAV, a well described/established recombinant viral vector for gene therapy, is the limited size of genome that could be packaged [98]. The size of wild type AAV genome is 4.7kb and packaging capacity is limited to this length [99]. It is believed that efficiency of packaging decreases as the length of the transgene increases [100]. As for the genetic disorders from the mutation in large genes such as Usher syndrome, development of a proper gene vector for viral gene therapy is still challenging. Since other viral vectors cannot insure the safety and efficacy (e.g., could result in mutagenesis or oncogene activation in case of retrovirus) [19], there were trials to overcome the size limitation of a recombinant AAV vector. One possible way is making two different transgenes; one can be an upstream gene and the other can be downstream. They could also be packaged in different strategies: fragmented, overlapping,* trans*-splicing, and hybrid [98].

Successful animal studies would enable the planning of clinical applications of gene and stem cell therapies in hearing loss patients. However, there are several important issues to consider. These include potential side effects. The viral vectors used in the studies should be tested in detail for their safety, for example, to ensure that they do not change their phenotype by cross-acting with a similar virus and thereby result in a contagious outbreak. As for the genome editing approach, minimizing the off-target effects must be warranted before the actual clinical applications. Stem cells must be properly controlled and optimized to avoid unnecessary cell growth and, thus, a tumorous condition. Another consideration is the socioeconomic effectiveness of gene and stem cell therapies, which, so far, are very expensive due to the high price of stem cells and viral vectors and their genetic modification. Moreover, their effectiveness is still questionable. Finally, the application of these novel approaches clinically to treat hearing loss rests upon the very high probability of hearing restoration.

Despite these limitations, gene and stem cell therapies remain a tempting strategy in hearing loss research because they are the only options that may lead to HC regeneration in the mature mammalian cochlea. Along with the successful outcomes reported in the literature, there is an ongoing human clinical trial of Ad delivered atonal gene (https://clinicaltrials.gov/ct2/show/NCT02132130) using CGF166 which is a recombinant Ad5 serotype containing the human atonal transcription factor (Hath1). Given the recent advances in genetics and cell biology as well as methods of overcoming current obstacles, the ultimate goal in clinical application of novel therapy for hearing loss may not be far away.

## Figures and Tables

**Table 1:** Animal studies using gene therapies for genetic hearing loss model.

**Year**	**Country**	**Vector**	**Gene**	**Animal**	**Reference**
2012	US	AAV	*VGLUT3*	mouse (premature)	Akil et al.
2014	US	AAV	*Gjb2*	mouse (mature)	Yu et al.
2015	Japan	AAV	*Gjb2*	mouse (mature and premature)	Iizuka et al.
2015	US	AAV	*TMC1*	mouse (premature)	Askew et al.
2016	South Korea	AAV	*MsrB3*	mouse (in utero)	Kim et al.
2017	US	AAV	*USH3*	mouse (premature)	Geng et al.
2017	US	AAV	*USH1C*	Mouse (premature)	Pan et al.

AAV: adenoassociated virus; *VGLUT3*: vesicular glutamate transporter 3; *MsrB3*: methionine sulfoxide reductase B3; *TMC1*: transmembrane channel like 1; *USH3: *Usher syndrome type III; *USH1C: *Usher syndrome type Ic.

**Table 2:** Animal studies using gene therapies for hearing loss protection (or hearing preservation).

**Year**	**Country**	**Vector**	**Ototoxic insults**	**Gene**	**Animal**	**Reference**
1999	US	Adv	Aminoglycoside	*GDNF*	Guinea pig	Yagi et al.
2004	US	Adv	Aminoglycoside	*SOD1*	Guinea pig	Kawamoto et al.
2006	US	AAV	Cisplatin	*XIAP*	rat	Cooper et al.
2008	Japan/China	AAV	Aminoglycoside	*GDNF*	rat	Liu et al.
2008	US	siRNA	Cisplatin	*TRPV1 and NOx3*	rat	Mukherjea et al.
2009	US	AAV	Aminoglycoside	*Bcl2*	mouse (mature)	Pfannenstiel et al.
2010	Australia	Adv	Aminoglycoside	*(NT3 and BDNF)*	Guinea pig	Wise et al.
2015	US	Adv	Aminoglycoside	*Hsp70*	Guinea pig	Takada et al.
2016	China	AAV	Cisplatin	*XIAP*	mouse (mature)	Jie et al.
2016	US	siRNA	Acoustic overexposure	*AMPK*	Mouse (mature)	Hill et al.

Adv: adenovirus; AAV: adenoassociated virus; *GDNF*: glial cell derived neurotrophic factor; *SOD*: superoxide dismutase; *XIAP*: X-linked inhibitor of apoptosis; *TRPV1*: Transient Receptor Potential Cation Channel Subfamily V Member 1; *NOx3*: NADPH Oxidase 3; *NT3*: neurotrophin 3; *BDNF*: brain derived neurotrophic factor; *Hsp70*: heat shock protein 70; *AMPK*: AMP-activated protein kinase.

**Table 3:** Animal studies using gene therapies for cochlear hair cell regeneration.

**Year**	**Country**	**Vector**	**Gene**	**Animal**	**Reference**
2003	US	Adv	*MATH1*	Guinea pig	Kawamoto et al.
2014	Australia	AAV	*ATOH1*	Guinea pig	Atkinson et al.

Adv: adenovirus; AAV: adenoassociated virus; GSI: gamma secretase inhibitor; GSI: gamma secretase inhibitor.
