# Corrigendum to “Potential of Gene and Cell Therapy for Inner Ear Hair Cells”

**DOI:** 10.1155/2019/9601260

**Published:** 2019-03-07

**Authors:** Min Young Lee, Yong-Ho Park

**Affiliations:** ^1^Department of Otorhinolaryngology and Head & Neck Surgery, Dankook University Hospital, Cheonan, Chungnam, Republic of Korea; ^2^Department of Otolaryngology-Head and Neck Surgery, College of Medicine, Chungnam National University, Daejeon, Republic of Korea; ^3^Brain Research Institute, College of Medicine, Chungnam National University, Daejeon, Republic of Korea

In the article titled “Potential of Gene and Cell Therapy for Inner Ear Hair Cells” [1], the name of the first author was given incorrectly as Min Yong Lee. The author's name should have been written as Min Young Lee. The revised authors' list is shown above.
